# *Porphyromonas gingivalis* PgFur Is a Member of a Novel Fur Subfamily With Non-canonical Function

**DOI:** 10.3389/fcimb.2019.00233

**Published:** 2019-07-01

**Authors:** Michał Śmiga, Marcin Bielecki, Mariusz Olczak, Teresa Olczak

**Affiliations:** Faculty of Biotechnology, University of Wrocław, Wrocław, Poland

**Keywords:** *Porphyromonas gingivalis*, ferric uptake regulation protein (Fur), PgFur, HmuY, transcription regulation, periodontitis

## Abstract

*Porphyromonas gingivalis*, a keystone pathogen of chronic periodontitis, uses ferric uptake regulator homolog (PgFur) to regulate production of virulence factors. This study aimed to characterize PgFur protein in regard to its structure-function relationship. We experimentally identified the 5′ mRNA sequence encoding the 171-amino-acid-long PgFur protein in the A7436 strain and examined this PgFur version as a full-length protein. PgFur protein did not bind to the canonical *Escherichia coli* Fur box, but the wild-type phenotype of the mutant Δ*pgfur* strain was restored partially when expression of the *ecfur* gene was induced from the native *pgfur* promoter. The full-length PgFur protein contained one zinc atom per protein monomer, but did not bind iron, manganese, or heme. Single cysteine substitutions of CXXC motifs resulted in phenotypes similar to the mutant Δ*pgfur* strain. The modified proteins were produced in *E. coli* at significantly lower levels, were highly unstable, and did not bind zinc. The *pgfur* gene was expressed at the highest levels in bacteria cultured for 24 h in the absence of iron and heme or at higher levels in bacteria cultured for 10 h in the presence of protoporphyrin IX source. No influence of high availability of Fe^2+^, Zn^2+^, or Mn^2+^ on *pgfur* gene expression was observed. Two chromosomal mutant strains producing protein lacking 4 (*pgfur*Δ*4aa*) or 13 (*pgfur*Δ*13aa*) C-terminal amino acid residues were examined in regard to importance of the C-terminal lysine-rich region. The *pgfur*Δ*13aa* strain showed a phenotype typical for the mutant Δ*pgfur* strain, but both the wild-type PgFur protein and its truncated version bound zinc with similar ability. The Δ*pgfur* mutant strain produced higher amounts of HmuY protein compared with the wild-type strain, suggesting compromised regulation of its expression. Potential PgFur ligands, Fe^2+^, Mn^2+^, Zn^2+^, PPIX, or serum components, did not influence HmuY production in the Δ*pgfur* mutant strain. The mutant *pgfur*Δ*4aa* and *pgfur*Δ*13aa* strains exhibited affected HmuY protein production. PgFur, regardless of the presence of the C-terminal lysine-rich region, bound to the *hmu* operon promoter. Our data suggest that cooperation of PgFur with partners/cofactors and/or protein/DNA modifications would be required to accomplish its role played in an *in vivo* multilayer regulatory network.

## Introduction

Periodontal diseases are among the most common human infections characterized by an auto-immune background. From the clinical point of view, periodontitis is characterized by deep periodontal pockets, resulting from the loss of connective tissue attachment and alveolar bone (Bostanci and Belibasakis, 2012; Tonetti et al., 2018). Recent reports also support their correlation with systemic diseases (e.g., Sudhakara et al., 2018; Albandar et al., 2018; Hegde and Awan, 2018; Dominy et al., 2019). Among several periodontopathogens, *Porphyromonas gingivalis* is considered the main etiologic agent and a keystone pathogen responsible for oral microbiome dysbiosis and chronic periodontitis development (Hajishengallis, 2011; Bostanci and Belibasakis, 2012; Lamont et al., 2018). *P. gingivalis* is not only a constituent of a multispecies biofilm formed in the oral cavity, but can also enter gingival epithelial and immune cells, and remain viable and capable of spreading among host cells (Lamont et al., 1995; Lamont and Yilmaz, 2002; Yilmaz et al., 2002, 2006; Guyodo et al., 2012; Olczak et al., 2015; Smiga et al., 2019).

To survive, replicate and efficiently infect the host, *P. gingivalis* produces a variety of virulence factors, which are under the control of regulatory mechanisms (Potempa et al., 2003; Aruni et al., 2013; Nakayama, 2015; Smiga et al., 2019). Regulation of gene expression in bacteria usually occurs at the transcriptional level by action of DNA-binding proteins (Hantke, 2001; Lee and Helmann, 2007). Among such proteins is the ferric uptake regulator (Fur), the main representative of the Fur superfamily, which can regulate expression of genes in response to iron availability through several different mechanisms (Pecqueur et al., 2006; Lee and Helmann, 2007; Fillat, 2014; Sarvan et al., 2018a,b). Taking into account the classical hypothesis based on a canonical functional mode, under high-iron conditions Fur becomes ferreted and binds to specific Fur box sequences located in promoters of regulated genes, which results in prevention of their transcription through steric hindrance of RNA polymerase (Hantke, 2001; Deng et al., 2015; Sarvan et al., 2018b). Under low-iron conditions the Fur-Fe^2+^ complex dissociates and the repressor becomes inactive, which leads to transcription of target genes. Fur regulates expression of many genes by direct or indirect mechanisms. For example, in *Neisseria meningitidis* Fur mediates classical iron-dependent repression of some genes (e.g., *tbp2*), while acting as an activator for other genes (e.g., *nor B*) (Delany et al., 2004). In contrast to iron-dependent DNA binding activity, some Fur proteins can sense signals other than iron, including heme, or bind to DNA in the absence of a metal cofactor (Dubrac and Touati, 2000; Hernandez et al., 2004; Butcher et al., 2012; Pellicer et al., 2012). In its apo-form, Fur is able to regulate target genes by repression (e.g., *Escherichia coli sodB*) (Dubrac and Touati, 2000) or by up-regulation (e.g., *Helicobacter pylori pfr*) (Delany et al., 2001). Therefore, it has been suggested that the up-regulation of some genes is linked to apo-Fur polymerization along the target DNA. In addition, a minor-groove readout mechanism used by Fur proteins has been recently proposed (Agriesti et al., 2014). Fur regulation also occurs through repression of sigma factors or two-component systems, which in turn regulate the expression of certain genes (Cornelis et al., 2009; Troxell and Hassan, 2013; Fillat, 2014; Yu et al., 2016). Importantly, it has been well-documented that Fur functions as a global regulator that controls not only the expression of iron acquisition systems, but also a large number of genes involved in different cellular processes, such as the stress response and production of virulence factors (Lee and Helmann, 2007). Interestingly, some bacteria, such as *Bacillus subtilis*, possess three Fur proteins that regulate the peroxide stress response, zinc uptake, and iron uptake (Ma et al., 2012).

Members of the Fur superfamily are composed of two regions: an N-terminal DNA-binding domain, containing a winged-helix motif, and a C-terminal dimerization domain, connected by a hinge loop (Lucarelli et al., 2007; Sheikh and Taylor, 2009; Dian et al., 2011; Butcher et al., 2012; Sarvan et al., 2018c). Fur from *Pseudomonas aeruginosa* possesses two zinc binding sites. Site 1 (S1) is present in the C-terminal dimerization domain and is hexacoordinated by two histidines, a glutamic acid, a bidentate aspartic acid and a water molecule. Site 2 (S2) is located in the hinge region between the DNA-binding domain and dimerization domain and exhibits a tetrahedral geometry with two histidines and two glutamates as ligands (Pohl et al., 2003). Another type of metal binding site present in the dimerization domain, occupied by zinc, was discovered in several purified Fur proteins, e.g., in Fur from *H. pylori* (Dian et al., 2011). However, despite numerous structural studies, the location and affinity of the metal binding site(s) and molecular mechanisms of gene regulation of Fur proteins are still controversial and very often unknown. Importantly, this area of research is poorly explored in *P. gingivalis*.

Previously, we characterized a Fur homolog (PgFur) identified in *P. gingivalis*. Our data showed that *pgfur* deletion mutant strain differed in gene expression, especially under iron/heme-depleted conditions, suggesting different or multiple molecular mechanisms of regulation of gene expression by PgFur (Ciuraszkiewicz et al., 2014; Smiga et al., 2019). We also demonstrated that PgFur regulated production of virulence factors differently in more (A7436) and less (ATCC 33277) virulent *P. gingivalis* strains (Smiga et al., 2019). The aim of this study was to characterize PgFur protein in regard to its structure-function relationship.

## Materials and Methods

### Bacterial Strains and Growth Conditions

*Porphyromonas gingivalis* wild-type (A7436), mutant (Δ*pgfur, pgfur*Δ4aa, *pgfur*Δ13aa, *pgfur* + HA), complemented mutant (Δ*pgfur* + *pgfur*, Δ*pgfur* + C102A, Δ*pgfur* + C107A, Δ*pgfur* + C110A, Δ*pgfur* + C148A, Δ*pgfur* + C151A, Δ*pgfur* + *ecfur*), and control (Δ*pgfur* + pTIO-tetQ) strains were grown anaerobically at 37°C for 5 days on blood agar plates composed of Schaedler broth (containing hemin and L-cysteine), and supplemented with 5% sheep blood and menadione (Biomaxima, ref. no. PP 0003) as described previously (Olczak et al., 2008). These cultures were used as the inoculum for growth in liquid basal medium (BM) composed of 3% trypticase soy broth (Becton Dickinson), 0.5% yeast extract (Biomaxima), 0.5 mg/l menadione (Fluka), and 0.05% L-cysteine (Sigma) as described previously (Ciuraszkiewicz et al., 2014; Smiga et al., 2019). To provide a source of protoporphyrin IX, BM was supplemented with 7.7 μM hemin (Fluka) (Hm), 7.7 μM protoporphyrin IX (PPIX) or 5% inactivated fetal bovine serum (Sigma) (FBS). To analyze the influence of chosen divalent ions on *P. gingivalis* growth and gene expression, BM was supplemented with 100 μM ZnCl_2_ (Zn^2+^), 100 μM FeSO_4_ (Fe^2+^), or 100 μM MnCl_2_ (Mn^2+^). To starve bacteria from iron and heme, cells were grown in the absence of heme and in the presence of an iron chelator, 160 μM 2,2-dipyridyl (DIP) (Sigma). *P. gingivalis* mutant strains were maintained in the presence of 1 μg/ml erythromycin. Complemented and control strains were maintained in the presence of 1 μg/ml erythromycin and 1 μg/ml tetracycline. Bacterial growth in liquid media was determined by measuring optical density at 600 nm (OD_600_) and on blood agar plates by visual inspection.

*Escherichia coli* Rosetta II (DE3) (Novagen) strain was grown under aerobic conditions in Terrific Broth (TB) with appropriate antibiotics as described previously (Bielecki et al., 2018).

### Construction of Modified *P. gingivalis* Strains

To determine the function of the C-terminus of the PgFur protein, mutant strains producing truncated PgFur protein or PgFur with addition of HA-tag at the C terminus were constructed. For this purpose, amplification of three DNA fragments was carried out by PCR with primers listed in Table 1. The obtained fragments were then ligated with NEBuilder HiFi DNA Assembly Kit (NEB), resulting in the DNA fragment comprising the *pgfur* gene encoding modified PgFur protein, *ermF* cassette (encoding the erythromycin resistance gene from *Bacteroides fragilis*) (Olczak et al., 2008), and DNA fragment flanking the 3′ end of the *pgfur* gene (Table 2). The entire DNA was subsequently amplified by PCR with primers listed in Table 1. Linear DNA fragments were used to electroporate the *P. gingivalis* A7436 strain (Simpson et al., 2000).

**Table 1:** Primers used in this study.

**Primer**	**5^**′**^ → 3^**′**^ DNA sequence (restriction sites are underlined)**	**Locus ID and/or gene abbreviation**	**Description or reference**
F1_FurHA_erm	ATGGTACCTTGGGCGATCTTT	PGA7_00014570 (*pgfur*), *ermF*, 3' DNA fragment flanking the *pgfur* gene	Amplify three fragments used for construction of modified *P. gingivalis* strains encoding PgFur + HA, PgFurΔ4aa, and PgFurΔ13aa. F1 and R1 amplify *pgfur* gene encoding modified PgFur protein; F2 and R2 amplify *ermF* cassette; F3 and R3 amplify 3′ DNA fragment flanking the *pgfur* gene. F1 and R3 were used to obtain the final fragments used for electroporation of *P. gingivalis* (this study and R3_fur_mut from Smiga et al., 2019).
R1_FurHA_erm	GTGTTAAGCATAATCTGGAACATCATATGGATATTTTTTCTT CTTGGGAGCGGCTTTG		
F2_FurHA_erm	AAATATCCATATGATGTTCCAGATTATGCTTAACACCGCGGTT GTCTCTCTTTC		
R2_FurHA_erm	GATCAATGTTATATGTCTGTGTTAGCCAGCCGTTATGCGG		
F3_ FurHA_erm	CGCATAACGGCTGGCTAACACAGACATATAACATTGATCC		
R3_fur_mut	CCACATTTGTCATTGACTGCTG		
R1_Fur167	GAAAGAGAGACAACCGCGGTGTTAGGGAGCGGCTTTGTCCG		
F2_Fur167	CGGACAAAGCCGCTCCCTAACACCGCGGTTGTCTCTCTTTC		
R1_Fur158	GAAGCAGTCGGCCCTCTAACACCGCGGTTGTCTCTCT		
F2_Fur158	GAGAGACAACCGCGGTGTTAGAGGGCCGACTGCTTCTT		
F_Fur_C102A	GCTTCATTTGCAGAGCAGGCTCCGCTGCTTTTCTGTACC		Primers used for introduction of point mutations into plasmids encoding *P. gingivalis* Fur (PgFur) protein (this study).
R_Fur_C102A	GGTACAGAAAAGCAGCGGAGCCTGCTCTGCAAATGAAGC		
F_Fur_C107A	AGTGTCCGCTGCTTTTCGCTACCGAATGTGCACAATTTTC		
R_Fur_C107A	GAAAATTGTGCACATTCGGTAGCGAAAAGCAGCGGACACTG		
F_Fur_C110A	CGCTGCTTTTCTGTACCGAAGCTGCACAATTTTCTACCTACTAC		
R_Fur_C110A	GTAGTAGGTAGAAAATTGTGCAGCTTCGGTACAGAAAAGCAGCG		
F_Fur_C148A	GCCTCTATGGTATAGCCGACAAATGCAGGAAGAAGCAG		
R_Fur_C148A	CTGCTTCTTCCTGCATTTGTCGGCTATACCATAGAGGC		
F_Fur_C151A	GGTATATGCGACAAAGCCAGGAAGAAGCAGTCGGC		
R_Fur_C151A	GCCGACTGCTTCTTCCTGGCTTTGTCGCATATACC		
F_EcFur	GTAGCTCCTGTCGATAGTGCCATGACTGATAACAATACCGCCCTAAAG	B21_RS03275(*ecfur*)	Amplify sequence used to exchange sequence encoding PgFur protein for EcFur protein in pTIO-tetQ + *pgfur* plasmid (this study).
F_EcFur	CGGATCAATGTTATATGTCTGTGTTATTATTTGCCTTCGTGCGCGTG		
F_pMALc5x_His	CCAACAAGGACCATAGATTATGCACCACCATCACCATCACC ATCACAAAATCGAAGAAGGTAAACTGG		Primers used for introduction of sequence encoding 6 His residues at the N-terminus of MBP in pMAL-c5x vector (this study).
F_pMALc5x_His	CCAGTTTACCTTCTTCGATTTTGTGATGGTGATGGTGATGGT GGTGCATAATCTATGGTCCTTGTTGG		
F_PgFur_blunt	ATGGCAGTTGTAAAGATGATAGTCAC	PGA7_00014570 (*pgfur*)	Amplify full length or 3' truncated end of the *pgfur* gene, used to clone into XmnI and BamHI cloning sites of pMAL-c5x_His plasmid (this study).
R_PgFur_BamHI	GACTGGATCCTTATTTTTTCTTCTTGGGAGCGGC		
R_PgFur158_BamHI	GACTGGATCCTTAGAGGGCCGACTGCTTCTT		
F_EcFur_blunt	ATGACTGATAACAATACCGCCCTAAAG	B21_RS03275 (*ecfur*)	Amplify ec*fur* gene used to clone into XmnI and BamHI cloning sites of pMAL-c5x_His plasmid (this study).
R_EcFur_BamHI	GACTGGATCCTTATTTGCCTTCGTGCGCGTG		
F_hmu_EMSA	CGGAATAATCGGCTGATACAC	PGA7_00004270 (*hmuY*)	Amplify DNA fragment of the *hmu* operon promoter used as a probe in EMSA; forward primer was biotinylated at the 5′ end (this study).
R_hmu_EMSA	TAGAGACACAATCAATGGCAATG		
GSP_RACE_fur	CCTTCATCGCCCCATTGCAG	PGA7_00014570(*pgfur*)	Primers used in 5′RACE experiments (this study).
NGSP_RACE_fur	GCGTGGTGGTCTGAGATCCTTGTCG		
NGSP2_RACE_fur	GGTAGAAAATTGTGCACATTCGGTAC		
rtFUR6F	TTCTGCGTTTGCCTTCTCC	PGA7_00014570(*pgfur*)	Amplify fragment of the *pgfur* gene used in RT-qPCR (Ciuraszkiewicz et al., 2014).
rtFUR6R	TGAGATCCTTGTCGGCCAGT		
16SrRNA-F	GCTTCGAAATACGAAACGT	PGA7_00000960,(*16S rRNA*)	Amplify fragment of the *16S rRNA* gene used in RT-qPCR (Gmiterek et al., 2013).
16SrRNA-R	TATATCCGTCTGTCGGAACG		

**Table 2:** Plasmids and *P. gingivalis* strains used in this study.

**Plasmid name**	**Description**	**References**
**PLASMIDS**		
pTIO-tetQ + *pgfur*	*pgfur*::*tetQ*	Smiga et al., 2019
pTIO-tetQ + C102A	*pgfur*C102A::*tetQ*	This study
pTIO-tetQ + C107A	*pgfur*C107A::*tetQ*	This study
pTIO-tetQ + C110A	*pgfur*C110A::*tetQ*	This study
pTIO-tetQ + C148A	*pgfur*C148A::*tetQ*	This study
pTIO-tetQ + C151A	*pgfur*C151A::*tetQ*	This study
pTIO-tetQ + *ecfur*	*ecfur*::*tetQ*	This study
pMAL_c5x		New England Biolabs
pMAL_c5x_His		This study
pMAL_c5x + *pgfur*		This study
pMAL_c5x + *pgfurΔ13aa*		This study
pMAL_c5x + *ecfur*		This study
**Strain name**	**Description**	**References**
***P. gingivalis*** **STRAINS**		
A7436	Wild-type	
Δ*pgfur*	A7436 strain with deleted *pgfur* gene	Ciuraszkiewicz et al., 2014
*pgfur*Δ4aa	A7436 strain producing *P. gingivalis* Fur (PgFur) protein lacking KKKK sequence at the C-terminus	This study
*pgfur*Δ13aa	A7436 strain producing PgFur protein lacking KKAADKAAPKKKK sequence at the C-terminus	This study
*Pgfur* + HA	A7436 strain producing PgFur protein with HA tag (YPYDVPDYA) at the C-terminus	This study
Δ*pgfur* + *pgfur*	Δ*pgfur* strain complemented with pTIO-tetQ + *pgfur* encoding wild-type PgFur protein	Smiga et al., 2019
Δ*pgfur* + C102A	Δ*pgfur* strain complemented with pTIO-tetQ + C102A encoding PgFur protein with Cys 102 replaced by Ala	This study
Δ*pgfur* + C107A	Δ*pgfur* strain complemented with pTIO-tetQ + C107A encoding PgFur protein with Cys 107 replaced by Ala	This study
Δ*pgfur* + C110A	Δ*pgfur* strain complemented with pTIO-tetQ + C110A encoding PgFur protein with Cys 110 replaced by Ala	This study
Δ*pgfur* + C148A	Δ*pgfur* strain complemented with pTIO-tetQ + C148A encoding PgFur protein with Cys 148 replaced by Ala	This study
Δ*pgfur* + C151A	Δ*pgfur* strain complemented with pTIO-tetQ + C151A encoding PgFur protein with Cys 151 replaced by Ala	This study
Δ*pgfur* + *ecfur*	Δ*pgfur* strain complemented with pTIO-tetQ + *ecfur* encoding wild-type *E. coli* Fur (EcFur) protein	This study
Δ*pgfur* + pTIO-tetQ	Δ*pgfur* strain complemented with pTIO-tetQ (control strain)	Smiga et al., 2019

To obtain strains encoding PgFur protein with single amino acid substitutions, appropriate modifications of pTIO-tetQ + *pgfur* plasmid (Smiga et al., 2019) were performed using the QuickChange II Site-Directed Mutagenesis Kit (Agilent Technologies) and primers listed in Table 1. The resulting plasmids (Table 2) were sequenced and used to transform *P. gingivalis* mutant Δ*pgfur* strain.

To obtain Δ*pgfur* + *ecfur* strain, modification of the pTIO-tetQ + *pgfur* plasmid was performed. The *ecfur* gene (B21_RS03275) was amplified with primers listed in Table 1 and introduced into the pTIO-tetQ + *pgfur* plasmid by cloning without the use of restriction enzymes, using the method developed by Bryksin and Matsumura (2010). The resulting pTIO-tetQ + *ecfur* plasmid (Table 2), comprising the native *pgfur* gene promoter and the DNA sequence encoding the EcFur protein, was sequenced and used to transform the *P. gingivalis* mutant Δ*pgfur* strain.

Homologous recombination between electroporated DNA and the chromosomal DNA of *P. gingivalis* and mutations introduced in the *pgfur* gene were verified by PCR and DNA sequencing (Microsynth AG) (data not shown).

### RT-qPCR

To determine relative changes in the *pgfur* gene expression, reverse transcription-quantitative PCR (RT-qPCR) was performed as described previously (Smiga et al., 2019). Briefly, RNA was isolated using the Total RNA Mini Kit and the Clean-Up RNA Concentrator Kit (A&A Biotechnology). cDNA was synthesized using the SensiFAST cDNA Synthesis Kit (Bioline). PCR was carried out using the SensiFAST SYBR No-ROX Kit (Bioline) and LightCycler 96 (Roche). To analyze the quality of PCR products, melting curves were generated. Relative quantification of genes was determined in comparison to the *16S rRNA* gene of *P. gingivalis* (*PGA7_00000960*) using the ΔΔC_t_ method. As a control, untranscribed RNA was used for exclusion of genomic DNA contamination. All samples were run in triplicate from two independent experiments. Primers used in this study are listed in Table 1.

The statistical analysis was performed using Student's *t-*test. Data were expressed as mean ± S.D. For statistical analysis, the GraphPad software (GraphPad Prism 5.0 Inc.) was used.

### Construction of Expression Plasmids, Overexpression, and Purification of Recombinant PgFur Protein Variants

pMAL-c5x + *pgfur*, pMAL-c5x + *pgfur*Δ*13aa*, and pMAL-c5x + *ecfur* plasmids were constructed using the pMAL-c5x vector (NEB). First, a DNA sequence encoding six histidine residues (6His) attached to the N-terminus of maltose binding protein (MBP) was added to the original pMAL-c5x vector using the QuickChange II Site-Directed Mutagenesis Kit and primers listed in Table 1, resulting in the pMAL-c5x_His plasmid. *pgfur* and *ecfur* genes were amplified by PCR and primers listed in Table 1, and subsequently cloned into pMAL-c5x_His XmnI and BamHI cloning sites. Plasmids encoding PgFur protein with single amino acid substitutions were prepared using the QuickChange II Site-Directed Mutagenesis Kit, pMAL-c5x + *pgfur* plasmid as a template, and primers listed in Table 1. The correctness of the cloned sequences was verified by DNA sequencing (Genomed).

*Escherichia coli* Rosetta II (DE3) strain (Novagen) was used for overexpression of PgFur protein variants. Respective plasmids were introduced into *E. coli* cells by heat-shock transformation and cultures were incubated at 37°C (220 rpm) until OD_600_ = 1 was reached. Protein overexpression was induced using 0.5 mM IPTG and cultures were further incubated overnight at 16°C (220 rpm). Protein expression levels were analyzed using denaturing polyacrylamide gel electrophoresis (SDS-PAGE) as described previously (Ciuraszkiewicz et al., 2014). Bacterial cultures were then centrifuged (4,000 × *g*, 20 min, 4°C) and pellets stored at −20°C.

To purify proteins, bacterial pellets were re-suspended in cold 25 mM HEPES, pH 7.8, containing 300 mM NaCl, lysed by sonication (UP100H Hielscher Ultrasonics, 4°C), and centrifuged (30,000 × *g*, 20 min, 4°C). PgFur protein variants present in the soluble fraction of cell lysates were bound to amylose resin (NEB) and unbound proteins were washed out with 25 mM HEPES, pH 7.8, containing 1 M NaCl. Recombinant PgFur protein variants were released from the resin using 25 mM maltose in 25 mM HEPES, pH 7.8, containing 80 mM NaCl. Proteins were then transferred into 25 mM HEPES, pH 7.8, containing 80 mM NaCl and 5% glycerol using Amicon Ultra-15, 10,000 NMWL (Millipore). MBP was cleaved off in the presence of 2 mM CaCl_2_ using Factor Xa (NEB) and incubation for 16 h at 4°C. Then, NaCl concentration was increased to 300 mM. In order to separate the PgFur protein from the MBP protein, affinity chromatography using TALON resin (Clontech) was carried out. As the last step of purification, gel filtration was performed in 25 mM HEPES, pH 7.8, containing 300 mM NaCl and 5% glycerol using Sephadex G-75 resin. Fractions containing homogeneous PgFur protein were collected and stored at −20°C. Overexpression and purification of PgFurΔ13aa and EcFur was performed analogously. PgFur protein variants with single amino acid substitutions were purified in the form of MBP-PgFur fusions. Except the native protein (WT) and C102A variant, other protein variants, even in the form of MBP fusion proteins, were highly unstable. Overexpression of MBP alone was also observed in those samples.

Total protein concentration was determined using the modified Bradford method (Roti-Nanoquant, Roth) (Zor and Selinger, 1996).

### 5′RACE

To determine the 5′ upstream region of the transcribed mRNA sequence of the *pgfur* gene, the method of rapid amplification of cDNA ends (5′RACE) was employed using the SMARTer RACE 5′/3′ Kit according to the manufacturer's (TakaraBio) instructions and primers listed in Table 1. Briefly, total RNA was isolated from *P. gingivalis* cells grown in BM supplemented with 7.7 μM hemin (Hm), then cDNA was synthesized, and subsequently used to perform PCR. Final PCR products were cloned into the pJET1.2 plasmid (Thermo Fisher) and sequenced (Microsynth AG).

### SDS-PAGE and Western Blotting

Proteins were separated in 12% polyacrylamide gels using SDS-PAGE and visualized by Coomassie Brilliant Blue G-250 (CBB) or transferred onto nitrocellulose membrane (Millipore) and detected with anti-HmuY antibodies as described previously (Smiga et al., 2015).

### Electrophoretic Mobility Shift Assay (EMSA)

Binding of PgFur protein to DNA fragments was performed using the LightShift Chemiluminescent EMSA Kit (Thermo Scientific) according to the manufacturer's protocol. Biotin-labeled DNA fragments were generated by PCR using primers listed in Table 1. Purified biotin-labeled DNA (1 ng) was mixed with binding buffer with addition of 2.5% glycerol, 150 mM KCl, 5 mM MgCl_2_, and 50 ng/μl poly (dI^*^dC). Alternatively, various concentrations of manganese, purified PgFur/PgFurΔ13aa/EcFur protein, non-biotinylated DNA, and 10 mM sodium dithionite were added. Samples were incubated for 20 min at room temperature and separated on pre-run (30 min) 6% polyacrylamide gels for 1 h at 150 V in 0.25 × TBE buffer (25 mM Tris, 25 mM boric acid, 0.5 mM EDTA, pH 8.6), and 10 mM sodium dithionite. Subsequently, transfer onto a nylon membrane (Bionovo) was carried out in the presence of 1 × TBE buffer (300 mA, 30 min). Then, membranes were subjected to crosslinking for 10 min with UV radiation at 274 nm (50 Hz). Labeled nucleic acids were visualized using the Chemiluminescent Nucleic Acid Detection Module Kit (Thermo Scientific) and Chemidoc MP Imaging System (Bio-Rad). Each EMSA experiment was carried out independently three times.

### Determination of Metal Content

To identify metal ions bound to the PgFur, inductively coupled plasma–atomic emission spectroscopy (ICP–AES) was carried out using a Liberty 220 spectrometer (Varian) with standard pneumatic nebulizer as reported previously (Olczak et al., 2009). Determination of zinc content in the purified PgFur protein sample was carried out in 50 mM boric buffer, pH 9.1, containing 200 mM NaCl, 10% glycerol, 1 mM TCEP. Purified PgFur was also subjected to incubation for 20 min in the same buffer, but with addition of FeCl_2_ or MnCl_2_ (1:100 protein:metal molar ratio) and 10 mM dithionite, with subsequent removal of excess iron or manganese using ultrafiltration (Amicon Ultra-15; 10 kDa MWCO). All PgFur samples were examined at a final concentration of 10 μM.

Identification of structural zinc was based on the protocol described by Lee and Helmann (2006). Briefly, 70 μg of PgFur protein in a complex with MBP (for determination of structural zinc in PgFur variants with cysteine residues replaced by an alanine residue) or proteins in a complex with MBP, but digested with Xa factor (experiments with PgFur, PgFurΔ13aa, and EcFur) were incubated for 5 min at room temperature with Laemmli loading buffer without reducing agent and 0.2% SDS. The protein was separated by SDS-PAGE as described previously (Bielecki et al., 2018). Then, the gel was incubated for 5 min in 500 μM 4-(2-pyridyl) resorcinol solution (PAR) (Sigma Aldrich) in 25 mM HEPES, pH 8.0, containing 100 mM NaCl and 10% glycerol, followed by incubation in the presence of 50 mM H_2_O_2_. Zinc-binding proteins appeared as orange bands. All proteins were subsequently stained in the gel with CBB G-250.

### Phylogenetic Analysis

To compare the sequences of proteins exhibiting homology to the PgFur protein, the Basic Local Alignment Search Tool (BLAST) was used. The comparison of amino acid sequences and preparation of the phylogenetic tree for PgFur protein and other members of the Fur superfamily were performed using the Clustal Omega and Simple Phylogeny tools from the EMBL-EBI server (the European Bioinformatics Institute). The EvolView server (Zhang et al., 2012; He et al., 2016) was used to visualize phylogenetic distances and classify protein representatives from the Fur superfamily. To graphically represent the comparison of amino acid sequences of proteins, the BoxShade tool from the ExPASy server was used. To determine the theoretical physicochemical properties of the PgFur protein, the ProtParam tool from the ExPASy server (Artimo et al., 2012) was used.

### Three-Dimensional Modeling of the PgFur Structure

Modeling of the three-dimensional PgFur protein structure was performed using the I-TASSER server (Zhang, 2008; Roy et al., 2010; Yang et al., 2015) with automatic assessment and selection of templates. Resulting models were analyzed using the SAVES v5.0 server, consisting of multiple programs for protein structure analysis [VERIFY3D (Bowie et al., 1991; Lüthy et al., 1992), ERRAT (Colovos and Yeates, 1993), PROVE (Lüthy et al., 1992; Hooft et al., 1996; Pontius et al., 1996), PROCHECK (Laskowski et al., 1993, 1996), and WHATCHECK (Vriend, 1990; Hooft et al., 1996; Vriend et al., 1997)]. The best model was selected and submitted to ModRefinement (Xu and Zhang, 2011) and COACH (Yang et al., 2013a,b) servers for structure refinement and determination of potential ligand binding, respectively. The resulting protein model was visualized using PYMOL software (The Open-Source PyMOL Molecular GraphicsSystem Version 0.99r6 Schrodinger, LLC Schrodinger, New York, NY, USA).

## Results and Discussion

### Identification of Functional Version of the *P. gingivalis* Protein (PgFur)

Recently, we demonstrated that expression of proteins involved in iron and heme uptake, namely some hemagglutinins, hemolysins, proteases, two component systems, and *hmu* operon genes, that are among the most important *P. gingivalis* virulence factors, was regulated by the PgFur protein (Ciuraszkiewicz et al., 2014; Smiga et al., 2019). However, we (Ciuraszkiewicz et al., 2014; Smiga et al., 2019) and others (Butler et al., 2014, 2015; Anaya-Bergman et al., 2015) demonstrated that in contrast to classical Fur proteins, putative *P. gingivalis* Fur homolog does not regulate expression of genes whose products are involved in global maintenance of iron homeostasis. This was not an unexpected finding since a similar effect has been observed for Fur homologs characterized in some other microorganisms (Wexler et al., 2003; Friedman and O'Brian, 2004; Yang et al., 2006; Johnston et al., 2007).

**Figure 1:**
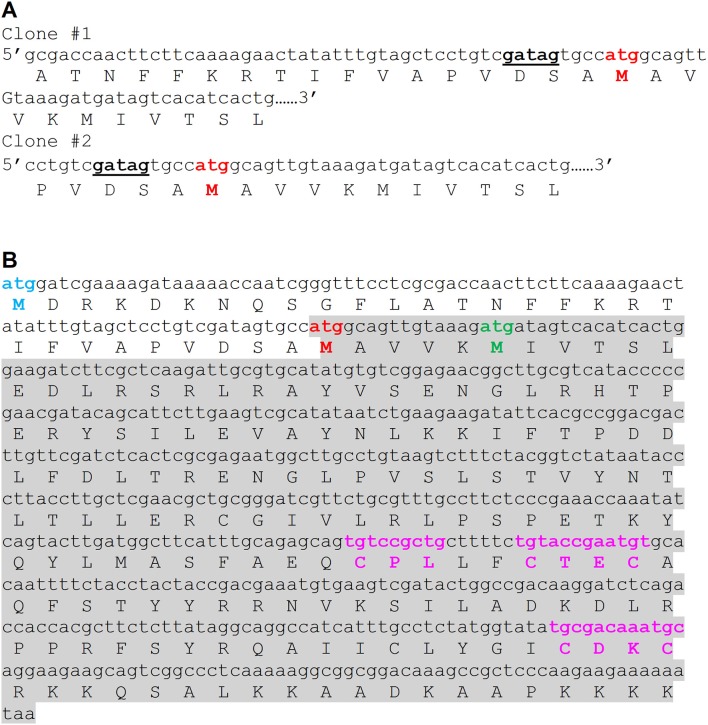
Comparison of amino acid sequence of the functional version of the PgFur protein identified experimentally in this study in the *P. gingivalis* A7436 strain with its versions deposited in databases. **(A)** Native mRNA sequence encoding PgFur protein was identified using 5′RACE technique. DNA sequences of two representative clones (out of 30 5′RACE PCR products sequenced) demonstrating the 5′ end of mRNA corresponding to the N terminus of the PgFur protein are shown. The putative ribosome-binding site (RBS) is shown in bold and underlined. **(B)** Amino acid sequences of PgFur protein versions identified in *P. gingivalis* strains. Start codon of the longest protein version (MDR) deposited in databases is shown in blue (e.g., BAG34022 in ATCC 33277, EOA10344 in JCVI SC001, AKV64657 in A7436) (Naito et al., 2008; McLean et al., 2013; Chastain-Gross et al., 2015), the shortest protein version (MIV) deposited in databases is shown in green (e.g., AAQ65662 in W83, BAK25731 in TDC60) (Nelson et al., 2003; Watanabe et al., 2011), and the protein version identified in this study (MAV) is shown in red. The entire sequence of the PgFur protein and its mRNA identified in this study is shadowed. Conserved, metal-binding CPL and CXXC motifs are shown in purple.

In this study, we attempted to investigate PgFur in regard to its structure-function relationship. To date, inconsistent amino acid sequences of a Fur homolog in *P. gingivalis* laboratory strains and clinical isolates identified based on genome sequencing have been deposited in databases. Among them are examples of 166-amino-acid-long proteins in W83 (AAQ65662) and TDC60 (BAK25731) (Nelson et al., 2003; Watanabe et al., 2011) and 200-amino-acid-long proteins in A7436 (AKV64657), ATCC 33277 (BAG34022), and JCVI SC001 (EOA10344) (Naito et al., 2008; McLean et al., 2013; Chastain-Gross et al., 2015; Figure 1). Experimental analysis performed by Butler et al. (2014) employed a 166-amino-acid-long version of the *P. gingivalis* Fur homolog (termed Har) but possessing the amino acid sequence derived from the ATCC 33277 strain. In this study, we experimentally identified the 5′ upstream region of mRNA encoding PgFur protein (Figure 1A) in the A7436 strain (GenBank accession number MK599280) and found that the functional protein is composed of 171 amino acid residues (19.7 kDa) (Figure 1B). We identified putative ribosome-binding site (RBS), namely GATAG sequence, suggesting that protein translation may start at the atggcagttgtaaag sequence (Figure 1A). However, it should be noted that high diversity of RBS in prokaryotic genomes exists and very little is also known about these sequences in species from phylum Bacteroidetes (Omotajo et al., 2015).

### PgFur and Classical Fur Proteins—Theoretical and Experimental Comparison

**Figure 2:**
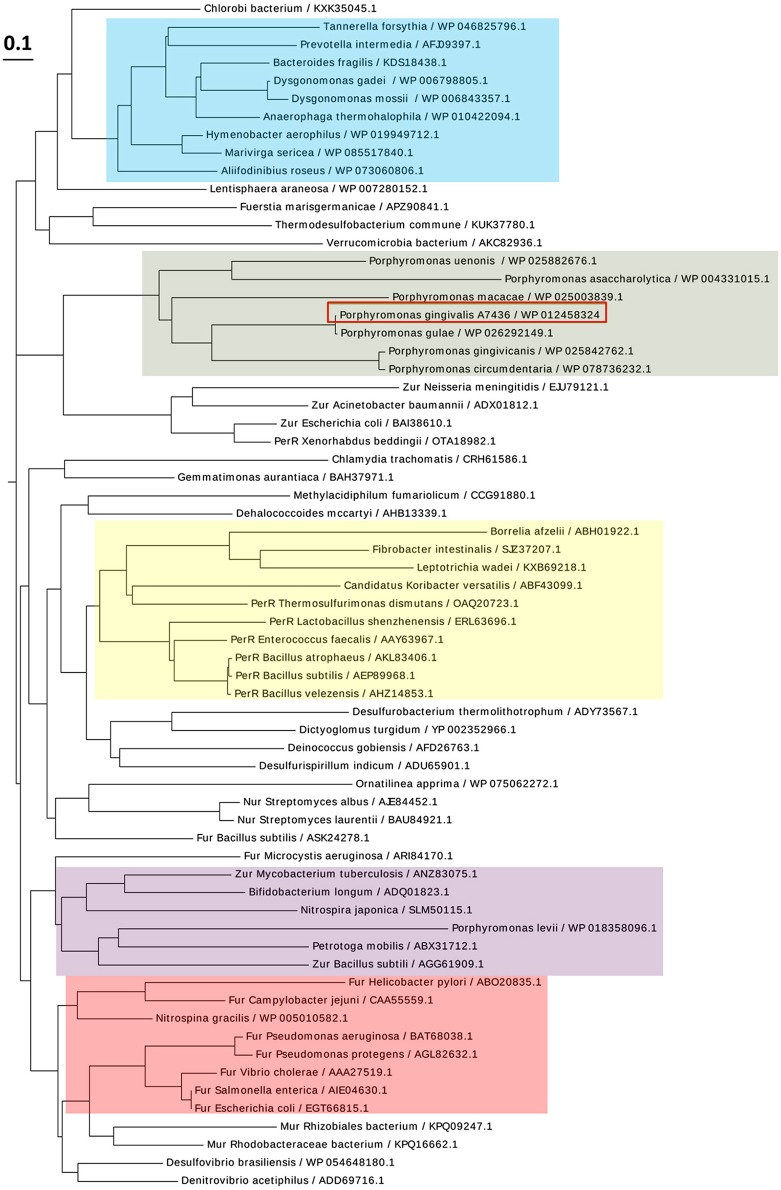
Diagram showing the phylogenetic distance of PgFur protein and selected representatives from the Fur superfamily. Protein classes are indicated with colors: green—homologs of Fur proteins found in *Porphyromonas* species, blue—homologs of Fur proteins identified in the phylum Bacteroidetes, red—classical Fur proteins, purple—Zur proteins, yellow—PerR proteins. Proteins are represented by the name of the subgroup (if assigned), the bacterial species name, and the NCBI ID number. The amino acid FASTA sequences were compared using the Clustal Omega server and Simple Phylogens. The phylogenetic tree was depicted using the EvolView server.

**Figure 3:**
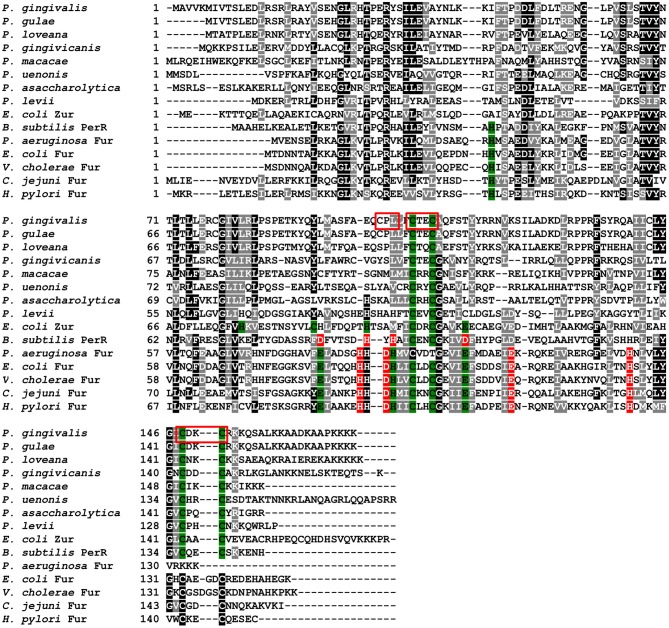
Analysis of amino acid sequences of PgFur protein and selected representatives of the Fur superfamily. Amino acid residues involved in coordination of metal ions are shown in red for Fe^2+^ and green for Zn^2+^. Analyzed motifs involved in metal binding by PgFur are shown in frames. Amino acid sequences of proteins identified by FASTA were compared using the Clustal Omega server. The drawing was generated using the BoxShade server.

PgFur amino acid sequence exhibits low identity to classical and best-characterized Fur proteins (Figures 2, 3). Therefore, in this study we focused on comparison between classical Fur superfamily members and their Fur homologs identified in the phylum Bacteroidetes. As shown in Figure 2, PgFur protein and all but *P. levii* Fur homologs found in *Porphyromonas* species are grouped together, forming a separate, distinct Fur subfamily compared with other representatives of the phylum Bacteroidetes, namely *Bacteroides fragilis, Prevotella intermedia*, and *Tannerella forsythia*, as well as with classical Fur, Zur, and PerR proteins. It is worth noting that even between *Porphyromonas* species and other Bacteroidetes members the amino acid identity was low (for example 28.57% between homologs identified in *P. gingivalis* and *B. fragilis*). Moreover, PgFur lacks characteristic motifs, found in the majority of other Fur superfamily members, rich in histidines involved in binding of regulatory metal ions (Figure 3). However, this is not surprising, since a similar feature was also ascribed to some representatives of Fur subfamilies, such as to Zur proteins (Feng et al., 2008).

**Figure 4:**
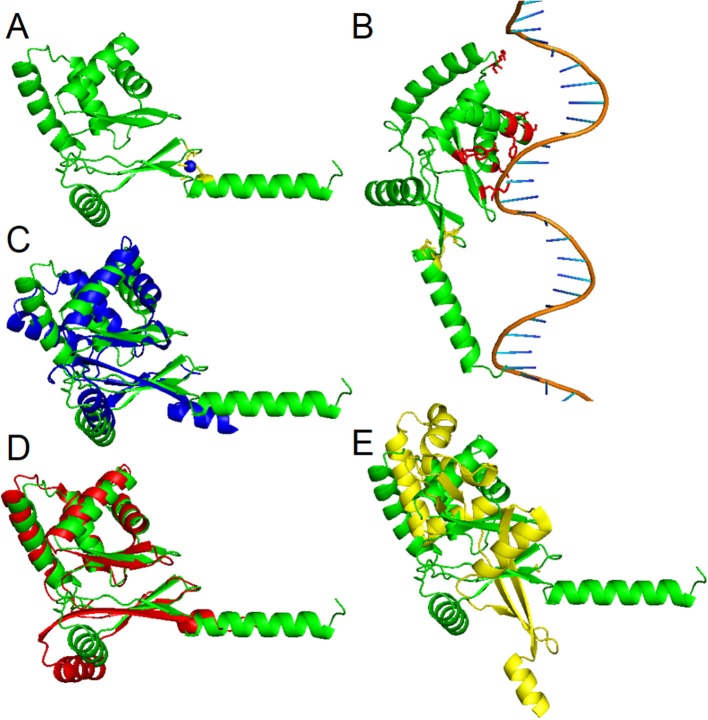
Homology modeling of the PgFur three-dimensional structure. **(A)** Theoretical PgFur tertiary structure in complex with zinc and **(B)** bound to DNA. Zinc-coordinating cysteine residues are shown in yellow, zinc ions in blue, and potential DNA binding amino acid residues in red. Comparison of PgFur theoretical structure (green) with experimentally identified protein structures of **(C)** *E. coli* Zur (PDB ID: 4MTD; blue), **(D)** *H. pylori* Fur (PDB ID: 2XIG; red), and **(E)** *B. subtilis* Per (PDB ID: 2FE3; yellow).

To date, three-dimensional structures have been reported for Fur proteins from *P. aeruginosa* (PDB ID: 1MZB) (Pohl et al., 2003), *Vibrio cholerae* (PDB ID: 2W57) (Sheikh and Taylor, 2009), *H. pylori* (PDB ID: 2XIG) (Dian et al., 2011), *Campylobacter jejuni* (PDB IDs: 4ETS, 6D57) (Butcher et al., 2012; Sarvan et al., 2018b), *Francisella tularensis* (PDB ID: 5NHK) (Pérard et al., 2018), and for a DNA-binding domain from *E. coli* Fur (PDB ID: 2FU4) (Pecqueur et al., 2006). A recent study showed the structure of Fur homolog from *Magnetospirillum gryphiswaldense* MSR-1 in a complex with manganese and *E. coli* Fur box (PDB ID: 4RB1) (Deng et al., 2015). To analyze the three-dimensional structure of the PgFur protein, a computational approach was employed using amino acid sequences of PgFur and its homologous proteins belonging to the extensive Fur superfamily. The theoretically modeled three-dimensional structure of the PgFur protein was constructed using experimentally solved protein structures of Fur superfamily members as templates. The PgFur protein model exhibits the highest similarity to Zur from *E. coli* (PDB ID: 4MTD), Fur from *H. pylori* (PDB ID: 2XIG) (Dian et al., 2011), and PerR from *B. subtilis* (PDB ID: 2FE3) (Figures 4A,C–E). All examined proteins share similar overall tertiary structure consisting of two domains: N-terminal domain engaged in DNA recognition and C-terminal domain involved in protein dimerization. However, templates used for this construction are not identical and comprise two different folds: the classical fold present in protein structures of *H. pylori* Fur (PDB ID: 2XIG) and *E. coli* Zur (PDB ID: 4MTD), and the alternative fold present in protein structures of *B. subtilis* PerR (PDB ID: 2FE3) and *C. jejuni* Fur (PDB ID: 4ETS, 6D57). Most visible difference between these two folds could be observed in the arrangement of alpha and beta structures, as well as in orientation of N- and C-terminal domains within the hinge region (Figures 4C–E). Importantly, in contrast to classical Fur proteins PgFur possesses a unique C-terminal region, probably organized in the form of an alpha-helix, with a very positive charge, which could be responsible for different DNA-binding and/or protein dimerization compared with classical Fur proteins. Our theoretical PgFur analysis showed potential zinc-binding sites (two CXXC motifs) (Figure 4A). Based on the homology modeling of the PgFur and *E. coli* Zur (PDB: 4MTD), the COACH server also indicates some amino acid residues which could be potentially involved in DNA binding (Asp50, Ser66, Tyr69, Asn70, Arg83, Tyr91, Tyr93) (Figure 4B).

**Figure 5:**
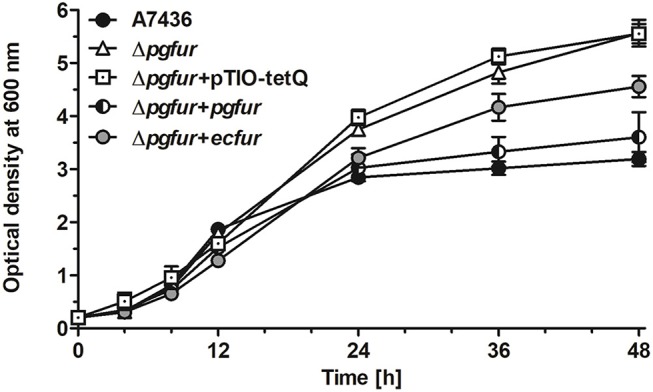
Comparison of growth rates of the wild-type (A7436), *pgfur* mutant (Δ*pgfur*), complemented mutant (Δ*pgfur* + *pgfur*, Δ*pgfur* + *ecfur*), and control (Δ*pgfur* + pTIO-tetQ) strains in iron/heme-rich medium (Hm). Experiments were performed three times from three biological replicates. Representative data are shown as mean ± standard deviation from one experiment performed in triplicate.

In our previous study, we reported that PgFur protein can partially complement the functional activity of *E. coli fur*-deletion mutant (Olczak et al., 2005). In this study we demonstrated that *E. coli* Fur (EcFur) protein partially complemented the lack of the active *pgfur* gene (Figure 5). The wild-type phenotype of the mutant Δ*pgfur* strain was restored partially when expression of the *ecfur* gene was induced under the control of the native *pgfur* promoter. However, we found that purified PgFur protein did not bind to the canonical *E. coli* Fur box (data not shown). These results further support significant differences between PgFur and classical Fur proteins.

### Structural and Regulatory Metals—Analysis of Metal Content and Its Influence on *pgfur* Gene Expression

Proteins belonging to the Fur superfamily can bind a variety of metal ions, which in turn regulates their DNA-binding activity; however, the mode of DNA recognition and binding for many of those proteins is still not clear. We found that the full-length PgFur protein version examined in this study, as isolated after purification, contained ~1 zinc atom per protein monomer (data not shown). This finding is in agreement with data reported for Har by Butler et al. (2014), as well as for other Fur proteins, for example for FurB from *Mycobacterium tuberculosis* (Lucarelli et al., 2007). However, in contrast to a study reported by Butler et al. (2014), we were not able to demonstrate binding of iron or manganese to the purified full-length protein (data not shown). Most of the Fur protein homologs from *Porphyromonas* species do not possess a motif rich in histidines responsible for regulatory metal binding (Figure 3). Although PgFur has one potential site which could be used to bind regulatory metal, namely CPL, such a motif is present only in *P. gingivalis* and *P. gulae*, but in the closely related *P. loveana* Fur homolog cysteine is substituted by serine (Figure 3), suggesting that it is very unlikely that the cysteine from the CPL motif could be involved in regulatory metal binding.

**Figure 6:**
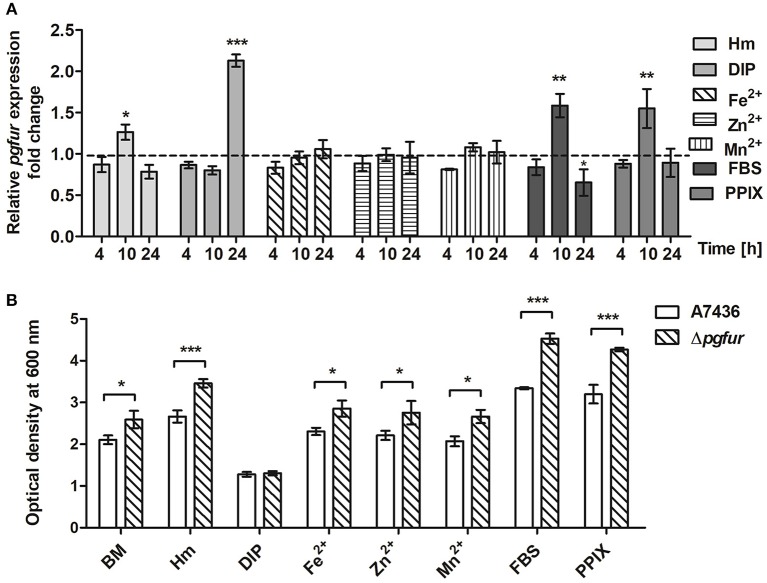
Changes in *pgfur* gene expression **(A)** and *P. gingivalis* growth rates under various culture conditions **(B)**. Expression of the *pgfur* gene was analyzed in respective culture media at the indicated time points in relation to the culture carried out in BM medium only **(A)**. The dashed line shows the gene expression under control conditions. *P. gingivalis* growth was determined after 24 h **(B)**. BM, no supplements; Hm, BM supplemented with 7.7 μM hemin; DIP, BM without hemin, supplemented with 160 μM dipyridyl; Fe^2+^, BM supplemented with 100 μM FeSO_4_; Zn^2+^, BM supplemented with 100 μM ZnCl_2_; Mn^2+^, BM supplemented with 100 μM MnCl_2_; FBS, BM supplemented with 5% FBS; PPIX, BM supplemented with 7.7 μM protoporphyrin IX. Experiments were performed three times from three biological replicates. Results are shown as mean ± standard deviation from two independent experiments performed in triplicate **(A)** or representative data are shown as mean ± standard deviation from one experiment performed in triplicate **(B)**. ^*^ *P* < 0.05; ^**^ *P* < 0.01; ^***^ *P* < 0.001.

To further examine the importance of potential metals for PgFur function, we compared *pgfur* gene expression in *P. gingivalis* grown under several conditions of ligand availability with that occurring in bacteria partly starved in BM, the latter mimicking those occurring in oral cavity of healthy individuals (low iron levels and lack of heme). As shown in Figure 6A, the *pgfur* gene was expressed at the highest levels when bacteria were cultured for 24 h in the most restricted conditions (absence of iron and heme; DIP). It is worth noting that these conditions are also characterized by low levels of other metals due to the chelating activity of dipyridyl. Higher levels of *pgfur* expression were also observed in bacteria grown for 10 h in BM supplemented with FBS or PPIX. In addition, in accordance with our previous findings, higher *pgfur* transcript levels were found in bacteria cultured in rich medium, conditions mimicking those occurring in the oral cavity of patients with chronic periodontitis (Hm) but also only for 10 h. The time points we have chosen to determine the *pgfur* gene expression show early exponential (4 h), middle exponential (10 h), and stationary growth phase (24 h). Bacteria in the exponential growth phase replicate at the highest rate, which means that the most accurate gene expression control is required. We think that higher *pgfur* gene expression rate is directly connected with bacteria replication rate which is the highest at 10 h. The highest replication rate is observed in the medium supplemented with heme, PPIX, and FBS, as a source of PPIX, which lack is the limiting factor for the growth of *P. gingivalis*. At certain time points of bacterial cultures replication stops and then bacteria are in the stationary phase. This is usually due to the utilization of nutrients and production of secondary metabolites. For regulation of this effect, the quorum sensing system is mostly involved. Therefore, we believe that *pgfur* deletion mutant strain encounters disturbance in the function of the quorum sensing system leading to higher growth rates in prolonged cultures. Some data supporting, at least in part, this assumption were published by us and by others previously (Butler et al., 2014; Ciuraszkiewicz et al., 2014). When bacteria starved in BM were subsequently cultured in BM in the presence of high availability of metals, no significant influence of an excess of Fe^2+^, Zn^2+^, or Mn^2+^ on *pgfur* gene expression was observed (Figure 6A). All but iron/heme-limited conditions (DIP) resulted in higher density of bacterial cultures of mutant Δ*pgfur* cells compared with the wild-type cells, especially after prolonged growth time (Figure 6B). Based on our results, we suggest that expression of the *pgfur* gene is dependent mostly on iron availability in the environment, which regulates its expression differentially at different growth stages.

In addition to the lack of iron or manganese binding, we were not able to demonstrate heme binding to the PgFur variant examined in this study (data not shown), which is in contrast to results reported for Har by Butler et al. (2014). One may assume that this effect might result from characterization of slightly different protein versions, resulting from examination of the full-length protein in the present study. In addition, in our recent report we showed that PgFur is less important for *P. gingivalis* virulence in the ATCC 33277 strain compared with the A7436 strain (Smiga et al., 2019), suggesting possibility of different PgFur expression in these strains. Based on our data we also assume that PgFur may function in apo-form, without a regulatory metal bound. This type of regulation within the Fur superfamily was reported for Fur from *H. pylori* and *C. jejuni*, as well as for Zur from *Corynebacterium glutamicum*, suggesting that it might be mediated by different orientation and conformation of DNA-binding domains in the apo-Fur protein dimers (Butcher et al., 2012; Teramoto et al., 2012; Agriesti et al., 2014). This hypothesis could be supported by growth analysis demonstrating higher cell density of *pgfur* mutant strain cultures measured after 24 h regardless of the conditions examined (BM, Hm, Fe^2+^, Zn^2+^, Mn^2+^, FBS, PPIX), except completely starved from iron and heme cultures (DIP) (Figure 6B).

**Figure 7:**
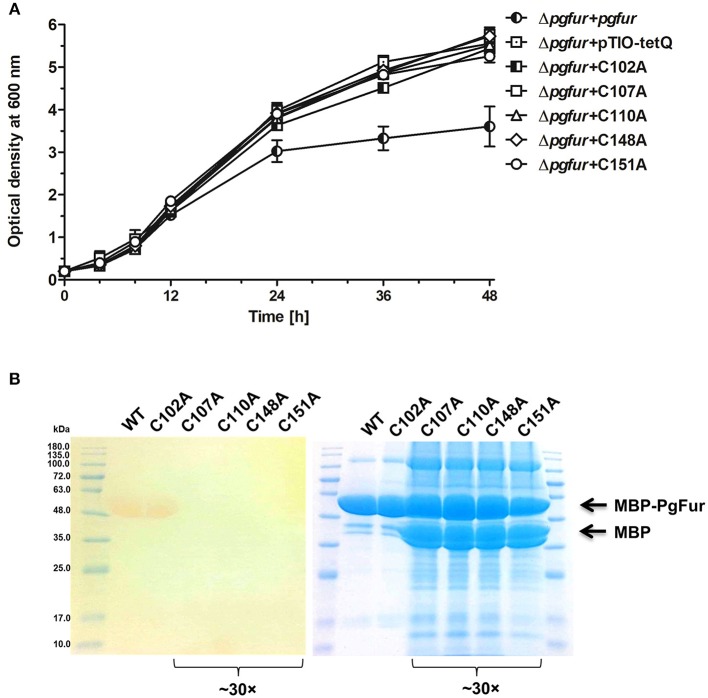
Identification of PgFur amino acid residues involved in metal binding. **(A)** Comparison of growth rates of complemented and control strains in iron/heme rich medium (Hm). Mutations introduced into the *pgfur* gene are shown in the strain's name. **(B)** Electrophoretic separation of proteins in polyacrylamide gel showing purified variants of the PgFur protein with maltose-binding protein (MBP) attached at the N-terminus, and their ability to bind Zn^2+^. The left panel shows zinc bound to proteins using PAR chelator. The right panel shows proteins stained with CBB G-250. The concentrated protein samples are indicated in the picture (~30×). Representative data are shown as mean ± standard deviation from one experiment performed in triplicate **(A)** or representative results out of three independent experiments are shown **(B)**.

PgFur does not possess histidines, which are important for metal binding in other Fur proteins (Figure 3). Therefore, we assumed that two CXXC motifs (C107TEC110 and C148DKC151) and one CPL motif (C102PL) could be involved in structural metal binding to the protein. Previously, Butler et al. (2014) showed that the CPL motif is not engaged in zinc binding to Har. To examine the importance of other cysteines for the binding of structural zinc, particular cysteine residues were substituted by alanine residues, and either phenotype of complemented mutant Δ*pgfur* strains was monitored or PgFur overexpressed and purified protein variants were analyzed in regard to Zn^2+^ binding. As shown in Figure 7A, only Δ*pgfur* + *C102A* exhibited a growth phenotype similar to the mutant Δ*pgfur* strain complemented with the native *pgfur* gene (Δ*pgfur* + *pgfur*). In contrast to this finding, single cysteine substitutions within two CXXC motifs resulted in phenotypes similar to the mutant Δ*pgfur* strain. Analysis of zinc binding to the purified protein variants demonstrated that only substitution of C102 resulted in similar zinc binding as compared to the unmodified purified protein (Figure 7B). In contrast, other modified proteins were produced in *E. coli* at significantly lower levels, the resulting proteins, even in the form of MBP fusion, both present in *E. coli* cell lysate and subsequently purified, were highly unstable, and did not bind Zn^2+^. These data confirm the importance of two CXXC motifs for structural zinc binding and subsequent proper protein folding.

### PgFur C-Terminal Amino Acid Sequence—Importance for Potential Function

**Figure 8:**
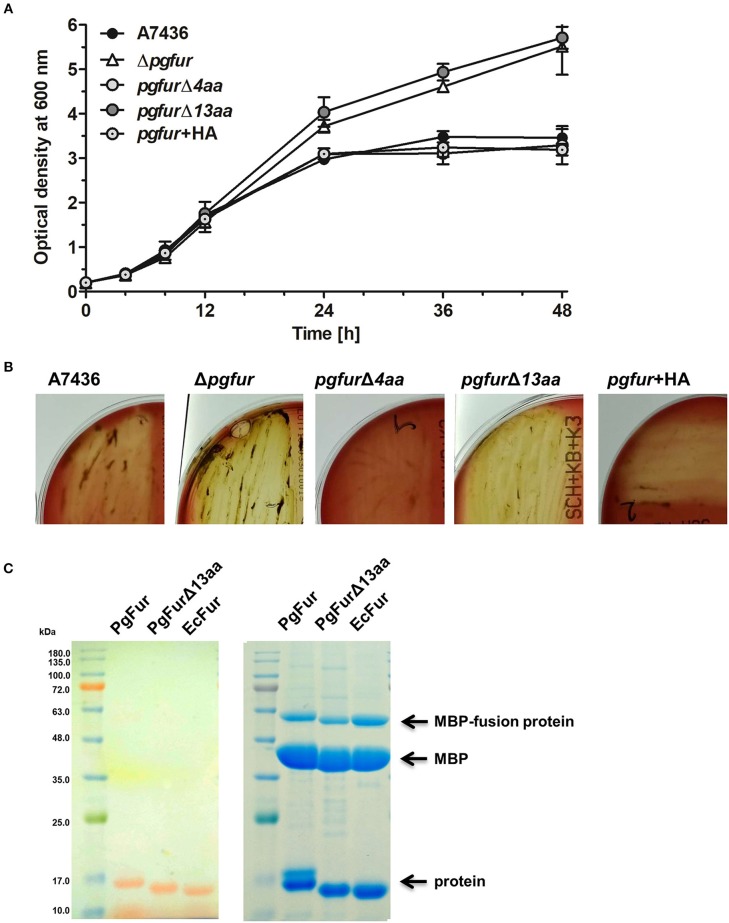
Characterization of the PgFur C-terminus. Comparison of growth rates in the iron/heme rich medium (Hm) **(A)** and hemolytic activity **(B)** of the wild-type (A7436) and *pgfur* mutant (Δ*pgfur*) strains, and strains producing modified PgFur protein (*pgfur*Δ*4aa, pgfur*Δ*13aa, pgfur* + *HA*). **(C)** Importance of the C-terminus for zinc binding. Purified full-length PgFur, PgFur lacking 13 C-terminal amino acid residues, and *E. coli* Fur (EcFur) were overexpressed, purified, and zinc bound to proteins was detected using PAR chelator (left panel). Proteins were subsequently stained with CBB G-250 (right panel). Representative data are shown as mean ± standard deviation from one experiment performed in triplicate **(A)** or representative results out of three independent experiments are shown **(B,C)**.

PgFur possesses an unusual C-terminal amino acid sequence, rich in lysine residues (Figures 3, 4), which results in alkaline character of the protein (theoretical pI = 9.47), whereas most of the proteins belonging to the Fur superfamily exhibit acidic character (e.g., *E. coli* Fur pI = 5.68, *E. coli* Zur pI = 5.97, *B. subtilis* PerR pI = 5.89, *C. jejuni* Fur pI = 7.05). To examine the influence of the C-terminus on potential PgFur function, we constructed two chromosomal mutant strains, allowing production of the protein lacking 4 (*pgfur*Δ*4aa*) or 13 (*pgfur*Δ*13aa*) C-terminal amino acid residues of the native PgFur protein. Taking into account growth ability in liquid cultures and hemolysis on blood agar plates (two most distinctive phenotypic features distinguishing the wild-type A7436 and mutant Δ*pgfur* strains) (Ciuraszkiewicz et al., 2014; Smiga et al., 2019), the *pgfur*Δ*4aa* strain, as well as the control strain producing PgFur protein with the HA epitope attached to the C-terminus of the protein, exhibited a phenotype similar to the wild-type strain (Figures 8A,B). In contrast, the *pgfur*Δ*13aa* strain showed a phenotype typical for the mutant Δ*pgfur* strain. We also showed that both the wild-type PgFur protein and its truncated version bound zinc with similar ability (Figure 8C). Efficient zinc binding was also observed for the purified EcFur protein, which was used as a positive control. This suggested that the unusual C terminus did not influence zinc binding, which is in agreement with results presented for Har by Butler et al. (2014).

During purification of the PgFur protein we noted high DNA binding capacity to the protein (data not shown). Therefore, we overexpressed, purified and analyzed the PgFur protein lacking 13 C-terminal amino acid residues to determine whether the KKAADKAAPKKKK region may be responsible for this property. A similar attempt was made by Butler et al. (2014), with the difference that the authors examined the 166-amino-acid-long protein derived from the ATCC 33277 strain and the protein C-terminally truncated by 16 amino acid residues. In our study, the truncated protein hardly showed interactions with DNA during the purification procedure, similar to the EcFur protein (data not shown). These data suggest that the C-terminal region might be important for the PgFur *in vivo* function, for example by increasing DNA-binding specificity and/or stability of the DNA-protein complex. Sequence-independent DNA binding due to the positively charged C-terminus was observed for other bacterial proteins (Szafran et al., 2014; Hołówka et al., 2017; Strzalka et al., 2017). Such interactions are important for stabilization of single-stranded DNA-protein interactions (e.g., *Streptomyces coelicolor* TopA) or for dynamic chromosome organization allowing efficient compaction of the nucleoid or effective transcription and translation (*Mycobacterium smegmatis* HupB).

### PgFur Function—Regulation of HmuY Expression

*P. gingivalis* uses several heme uptake systems, including that encoded by the *hmu* operon, comprising HmuR (a typical TonB-dependent receptor engaged in heme transport through the outer membrane), HmuY (a heme-binding hemophore-like protein), and four proteins of unknown function (Smalley and Olczak, 2017). The bacterium produces higher levels of HmuY under low-iron/heme conditions or when growing in the biofilm structure (Olczak et al., 2008, 2010), as well as inside host cells (Olczak et al., 2015). The HmuY protein is associated with the outer membrane of the bacterial cell and outer-membrane vesicles through a lipid anchor (Olczak et al., 2010; Veith et al., 2014), and can be processed into soluble form by limited proteolysis caused by *P. gingivalis* lysine-specific gingipain K (Kgp) (Wojtowicz et al., 2009; Olczak et al., 2010).

**Figure 9:**
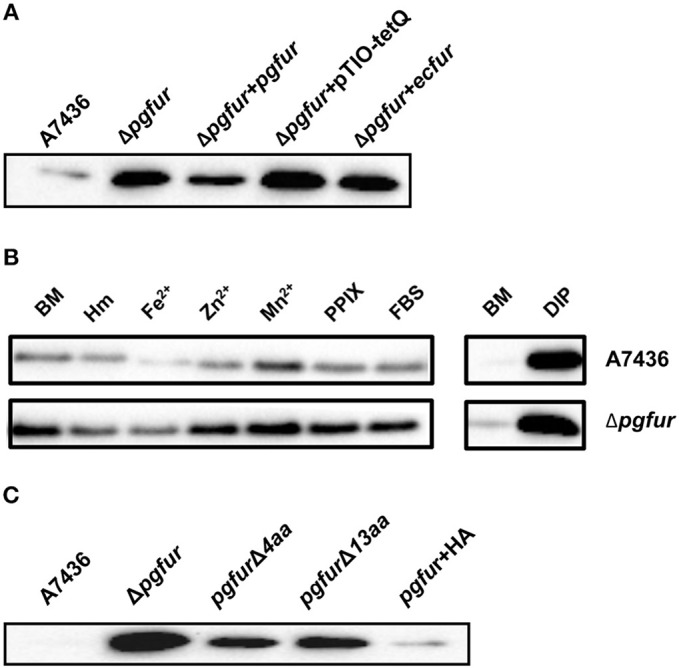
Involvement of PgFur in HmuY protein production. **(A)** HmuY protein levels in *P. gingivalis* wild-type (A7436), *pgfur* mutant (Δ*pgfur*), complemented mutant (Δ*pgfur* + *pgfur*, Δ*pgfur* + *ecfur*), and control (Δ*pgfur* + pTIO-tetQ) strains cultured in basal medium (BM) for 24 h. **(B)** HmuY produced by *P. gingivalis* strains in various culture media. **(C)** Production of HmuY protein by *P. gingivalis* strains producing modified PgFur protein. HmuY was detected with anti-HmuY antibodies and anti-rabbit-IgG antibodies conjugated with HRP using Western blotting. BM, no supplements; Hm, BM supplemented with 7.7 μM hemin; DIP, BM without hemin, supplemented with 160 μM dipyridyl; Fe^2+^, BM supplemented with 100 μM FeSO_4_; Zn^2+^, BM supplemented with 100 μM ZnCl_2_; Mn^2+^, BM supplemented with 100 μM MnCl_2_; FBS, BM supplemented with 5% FBS; PPIX, BM supplemented with 7.7 μM protoporphyrin IX. Representative results out of three independent experiments with a similar tendency are shown.

**Figure 10:**
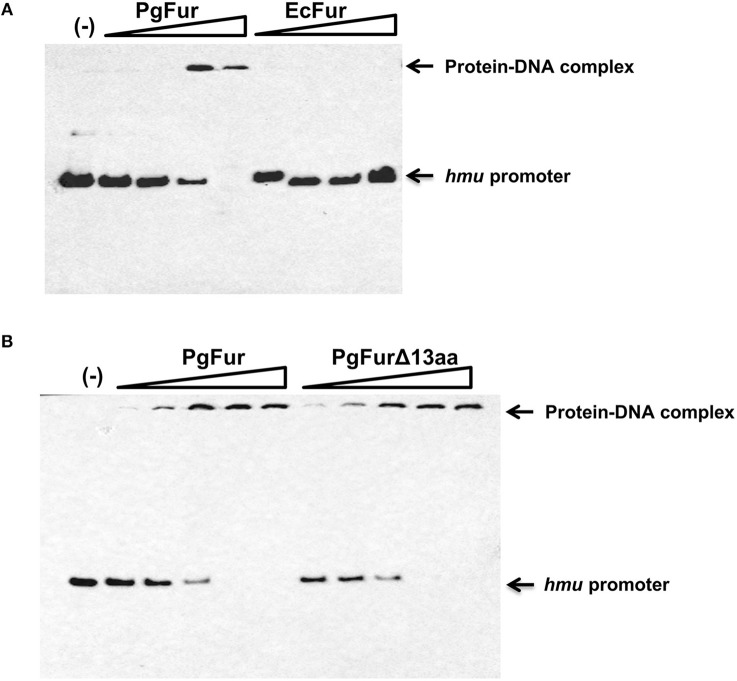
Binding of the PgFur protein to the *hmu* operon promoter. PgFur and EcFur **(A)**, or PgFur and PgFurΔ13aa **(B)** proteins were incubated with the *hmu* promoter fragment at a ratio of 0:1, 4:1, 10:1, 100:1, 1000:1, 10000:1 in the presence of Fe^2+^. Representative results out of three independent experiments with a similar tendency are shown.

Our previous reports demonstrated that PgFur might specifically and differently regulate expression of *hmu* operon genes, mainly *hmuY* gene (Ciuraszkiewicz et al., 2014; Smiga et al., 2019). Therefore, in this study we examined HmuY expression at the protein level in several modified *P. gingivalis* A7436 strains. As shown in Figures 9A,B, the partly starved (BM) mutant Δ*pgfur* strain produced higher amounts of HmuY protein compared with the wild-type strain. Complementation of the mutant strain with the native gene (Δ*pgfur* + *pgfur*) resulted in partial reversion of the mutant phenotype. Such an effect was not observed when the control strain harboring the pTIO-tetQ plasmid only or the plasmid encoding the *ecfur* gene was examined (Figure 9A). Analysis of *P. gingivalis* strains grown in cultures highly enriched with potential ligands demonstrated no significant influence of the examined components on HmuY protein production (Figure 9B). This conclusion was based on the similar HmuY production pattern observed in the case of all conditions examined. Importantly, we corroborated our data by demonstrating the potential importance of the C-terminal lysine-rich region for PgFur function. The modified *pgfur*Δ*4aa* and *pgfur*Δ*13aa* strains produced higher levels of HmuY protein, comparable to those observed for the Δ*pgfur* mutant strain (Figure 9C). These results support the involvement of the PgFur protein in regulation of *hmuY* gene expression, as well as the importance of the C-terminal region for proper PgFur *in vivo* function in the *P. gingivalis* A7436 strain. To further demonstrate regulation of the *hmuY* gene expression through the direct binding of the PgFur protein to the *hmu* promoter, EMSA analysis was employed. We found that PgFur did not bind to the classical Fur box derived from the EcFur promoter region (data not shown). However, as shown in Figure 10A, PgFur bound to the *hmu* operon promoter region, but surprisingly regardless of the presence of the C-terminal lysine-rich region. In contrast, no binding was observed when EcFur protein was examined (Figure 10B). Others demonstrated that *P. gingivalis* Fur homolog (Har) can also bind *in vitro* to the *dnaA* promoter region (Butler et al., 2014). This is not surprising because we (data not shown) and others (Butler et al., 2014; Anaya-Bergman et al., 2015) found that expression of this gene was regulated by a *P. gingivalis* Fur homolog. We hypothesize that PgFur binds specifically to the target region within regulated promoters (e.g., *hmuY, dnaA*), but also binds non-specifically to DNA, similar to other transcription factors, which utilize an intermediate step in the mechanism of sequence-specific recognition and binding to cognate sites (reviewed by Kalodimos et al., 2004). All these processes require recognition of steric and chemical features, which results in final binding and gene expression regulation. One may also speculate that PgFur might bind DNA *in vivo* as a multiprotein complex or require protein/DNA modification (e.g., acetylation, methylation) (reviewed by Slattery et al., 2014), which would modify its *in vivo* DNA-binding specificity.

## Conclusions

*Porphyromonas gingivalis* uses PgFur, a ferric uptake regulator homolog, to regulate production of virulence factors differently in more (A7436) and less virulent (ATCC 33277) strains (Smiga et al., 2019). In the current study we experimentally demonstrated that functional PgFur protein in the A7436 strain is 171 amino acids long. Although purified PgFur protein did not bind to the canonical *E. coli* Fur box, the wild-type phenotype of the mutant Δ*pgfur* strain was restored partially when expression of the *ecfur* gene was induced under the control of the native *pgfur* promoter. The full-length PgFur protein coordinated one zinc atom by four cysteine residues comprising two CXXC motifs, and did not bind iron, manganese or heme. The *pgfur* gene was expressed differentially depending on the growth phase and culture conditions. We further showed that PgFur could specifically regulate expression of the *hmuY* gene, at least in part through direct binding to the *hmu* promoter. We demonstrated that the unusual C-terminal lysine-rich region might be important for its *in vivo* DNA-binding specificity. Taken together, our data presented in this report confirm a different mechanism of PgFur function compared to canonical Fur proteins ascribed to the Fur superfamily members and support existence of a novel Fur subfamily, evolved in some *Porphyromonas* species, comprising keystone periodontopathogens of chronic periodontitis.

## Data Availability

The data for this study can be found in the GenBank database under accession number MK599280.

## Author Contributions

TO, MŚ, and MO designed the study. MŚ, MO, and MB performed the experiments. TO wrote the manuscript and provided financial support. All authors performed data analysis and approved the final version of the manuscript.

### Conflict of Interest Statement

The authors declare that the research was conducted in the absence of any commercial or financial relationships that could be construed as a potential conflict of interest.
